# Aggregation of M3 (E376D) variant of alpha1- antitrypsin

**DOI:** 10.1038/s41598-020-64860-1

**Published:** 2020-05-19

**Authors:** Arif Bashir, Younis Hazari, Debnath Pal, Dibyajyoti Maity, Samirul Bashir, Laishram Rajendrakumar Singh, Naveed Nazir Shah, Khalid Majid Fazili

**Affiliations:** 1 UPR Signalling Laboratory, Department of Biotechnology, University of Kashmir, Srinagar, 190006 Jammu and Kashmir India; 2 Department of Computational and Data Sciences (CDS), Indian Institute of Sciences, Bengaluru, 560012 India; 3 Dr. B. R. Ambedkar Center for Biomedical Research (ACBR), University of Delhi, Delhi, 110007 India; 4 Department of Chest Medicine, Govt. Medical College, Srinagar, 190001 Jammu and Kashmir India; 5 Present Address: Laboratory of Proteostasis Control and Biomedicine, Faculty of Medicine, University of Chile, Av. Independencia, 1027 Santiago Chile

**Keywords:** Biophysical chemistry, Computational models

## Abstract

Alpha1-antitrypsin (α1AT) is an abundant serine-protease inhibitor in circulation. It has an important role in neutralizing the neutrophil elastase activity. Different pathogenic point mutations like Z^(E342K)^-α1AT have been implicated in the development of liver cirrhosis and Chronic Obstructive Pulmonary Disease (COPD), the latter being a cluster of progressive lung diseases including chronic bronchitis and emphysema. M3-α1AT (376Glu > Asp) is another variant of α1AT which so far is largely being considered as normal though increased frequency of the variant has been reported in many human diseases including COPD. We also observed increased frequency of M3-α1AT in COPD cases in Kashmiri population. The frequency of heterozygous (AC) genotype in cases and controls was 58.57% and 27.61% (odds-ratio 6.53 (2.27–15.21); p < 0.0001) respectively, while homozygous CC genotype was found to be 21.42% and 6.66% (odds-ratio 10.56 (3.63–18.64); p < 0.0001) respectively. Comparative *in vitro* investigations that include trypsin‒antitrypsin assay, Circular Dichroism spectroscopy and dynamic light scattering performed on wild-type (M-α1AT), M3-α1AT, and Z-α1AT proteins along with the molecular dynamics simulations revealed that M3-α1AT has properties similar to Z-α1AT capable of forming aggregates of varied size. Our maiden observations suggest that M3-α1AT may contribute to the pathogenesis of COPD and other disorders by mechanisms that warrant further investigations.

## Introduction

Alpha1-antitrypsin (α1AT) is one of the most abundant circulating antiproteases. The serum levels of α1AT are raised secondary to activation of inflammatory-immune processes in humans^1^. α1AT is coded by a serine-protease inhibitor (*SERPIN*) A1. It is primarily expressed in hepatocytes and to some extent by lung tissue, macrophages, and monocytes. Among the different variants of α1AT, Z-α1AT (Glu342Lys) is the most pathogenic variant of α1AT and has been extensively studied. This variant has a distinctive capacity to form loop-sheet polymers due to the conformational change. The gain-of-toxic function of Z-α1AT in the hepatocytes leads to the manifestation of cirrhosis and hepatocellular carcinoma. On the contrary, reduced levels of serum α1AT lead to unregulated neutrophil elastase activity leading to the pathogenesis of a host of diseases including emphysema^1^. Owing to its pathogenicity, Z-α1AT is considered as a double-edged sword whose aggregation, on one hand leads to a pathological state of liver and loss-of-function on the other side results in emphysema^2^. The X-ray crystallography, *in silico* analysis, and kinetics of α1AT have provided valuable insights to understand its folding mechanism^3,4^. The conformational plasticity of serpins is not only important in terms of inhibitory activity but also unfolds a mechanistic understanding of their susceptibility towards misfolding and aggregation. During the folding process, α1AT is kinetically trapped in a metastable state. In this state, a patch of 15 amino acid residues (345–360) located near the C-terminus of α1AT, protruding out of its main body, is exposed to the polar solvent as a flexible loop connected between β-s5A and β-s1C that is called as reactive center loop (RCL). Native fold of α1AT is composed of three β-sheets (A–C) surrounded by 8–9 α-helices (hA–hI). The interaction of protease with the metastable α1AT gives rise to a marked conformational transition driven upon cleavage at P1′‒P1 site in the RCL.

The 342 Glu^−^ → Lys^+^ substitution just above the top of s5A strand in Z-α1AT removes a salt bridge between Glu342 and Lys290, thereby driving an electrostatic repulsion between them. This promotes polymerization by delaying the already slow insertion of s5A that prolong the exposure of the C-terminal domain of Z-α1AT^4^. Z-α1AT is the commonest of all the deficient variant observed with serum levels 0.06–0.2 g/L among homozygotes^5–7^. In contrast, the normal level of serum α1AT is 1.0‒3.6 g/L (catalogue number *ab108798*-α1AT human ELISA kit, *Abcam*), and the S (Glu264Val) variant of α1AT, frequent in the Mediterranean area, has minor reductions in serum α1AT levels (0.4–0.9 g/L). Many investigators consider M3-α1AT variant (1200 A > C, 376Glu → Asp) as good as wild-type with the normal levels of serum α1AT^8^. On the contrary, investigations, based purely on epidemiological data, have revealed its increased frequency in patients suffering from Guillain-Barre syndrome, chronic hepatitis, chronic inflammatory demyelinating polyneuropathy, and multiple sclerosis^9^. An investigation has reported three times higher frequency of PIM3 allele in Alzheimer’s patients and another investigation has reported an association of M3-α1AT allele with COPD^10,11^.

An increased frequency of M3-α1AT genotype in COPD patients and significantly lower levels of serum α1AT in both heterozygous (AC) and homozygous (CC) individuals of this variant genotype compared to a normal variant of α1AT gene has also been reported^12^. However, no investigation has been done to understand how this variant could possibly play its role in the development of a host of diseases including COPD. Our study reveals a significant association of M3-α1AT variant with the COPD in Kashmiri population. A study has reported 80% of frequency of M3 variant in COPD cases compared to control (37%)^12^. S (Glu264Val), the deficiency variant of α1AT, is more frequent in the Mediterranean area and is associated with minor reductions in serum AAT levels (0.4–0.9 g/L). Z variant of α1AT is the common deficient variant with serum levels 0.06–0.2 g/L among homozygotes. In our previous study, the mean serum α1AT level in COPD cases carrying M3 variant on a promoter hepatocyte background (composite heterozygote) was found to be 1.20 ± 0.24 and 3.16 ± 0.16 g/L in cases and controls, respectively. The serum α1AT level in COPD cases was found to be 2.6 times less than the control group^13^. In this investigation, we explored this association further in context of M3-α1AT being largely considered as a normal variant of α1AT. We generated M3-α1AT and Z-α1AT through site-directed mutagenesis. The wild-type M-α1AT and its variants (M3-α1AT, and Z-α1AT) were expressed in *E. Coli* BL21-DE bacterial expression system. All the purified α1AT proteins were used for further studies. Our biochemical and biophysical studies coupled with *in silico* analysis led us to conclude that M3-α1AT cannot be considered as a normal variant of α1AT and could possibly play a key role in the pathogenesis of a host of diseases including COPD that warrants further insight.

## Results

### High prevalence of M3 variant in COPD patients

The frequencies of genotypes of *SERPINA1*-exon5 376A/C SNP for both the COPD cases and controls are listed in Table 1. The overall association between the *SERPINA1*-exon5 376A/C SNP and the modulation of COPD risk was found to be statistically significant (p < 0.0001). The numbers and the frequencies of the subsets of various characteristics of the COPD cases under study like gender, dwelling, smoking status, hemoptysis, obstructive jaundice, and family history for this SNP are listed in Table S1.

**Table 1:** *SERPINA1*-exon5 376A/C single nucleotide polymorphism genotype frequency distributions among COPD cases and healthy controls and risk of COPD.

Genotype	COPD cases (N = 70)*	Controls (N = 105)*	OR (95% CI); p-value^#^	Adjusted OR^a^ (95% CI); p-value^#^	χ^2^; Pearson p-value (overall)^#,b^
AA	14 (20.00%)	69 (65.71%)	**1.0 (Reference)**	**1.0 (Reference)**	**35.85;** **p** < **0.0001**
AC	41 (58.57%)	29 (27.61%)	**6.96 (3.30‒14.68);** **p** < **0.0001**	$$\begin{array}{c}{\bf{6}}.{\bf{53}}({\bf{2}}.{\bf{27}}\mbox{--}{\bf{15}}.{\bf{21}});\\ {\bf{p}} < {\bf{0}}.{\bf{0001}}\\ {\bf{9}}.{\bf{21}}({\bf{3}}.{\bf{81}}\mbox{--}{\bf{29}}.{\bf{37}});\\ {\bf{p}} < {\bf{0}}.{\bf{0001}}\end{array}\}$$	
CC	15 (21.42%)	07 (06.66%)	**10.56 (3.63‒30.64);** **p** < **0.0001**		
A/C + C/C	56 (80.00%)	36 (34.28%)	**7.66 (3.76‒15.60);** **p** < **0.0001**	**7.81 (2.45–14.53);** **p** < **0.0001**	**p** < **0.0001**
**Allele**					
A	69 (49.28%)	167(79.52%)	**1.0 (Reference)**		
C	71 (50.71%)	43 (20.47%)	**3.99 (2.49‒6.40);** **p** < **0.0001**	—	**34.97;** **p** < **0.0001**

*N denotes the number of subjects or individuals. ^#^The values in bold indicate significant results. COPD, chronic obstructive pulmonary disease; OR, odds ratio. CI, confidence interval; ORs (95% CIs) were obtained from conditional logistic regression models. ^a^Adjusted ORs (95% CIs) were obtained in conditional logistic regression models when adjusted for age, gender, place of residence and smoking status. ^b^p-values calculated using χ^2^-tests.

The increased frequency of the variant genotypes (AC and CC) in COPD cases compared to controls in ours and other previous studies led us to hypothesize that M3-α1AT is not a normal variant and somehow contribute to the pathogenesis of COPD and other diseases as well. To understand the underlying mechanism, we cloned the wild-type (M-α1AT) in the bacterial expression vector (pET30a). We also generated our variants of interest (Z-α1AT and M3-α1AT) from M-α1AT through site-directed mutagenesis. All the α1AT proteins were purified to homogeneity to perform biochemical, and biophysical analysis. We compared the results of M3-α1AT against the Z-α1AT and M-α1AT. The wet lab results were further substantiated by performing a deep one-microsecond molecular dynamics simulations of all the α1AT proteins under consideration.

### All the α1AT proteins were found in a dimeric form

**Figure 1:**
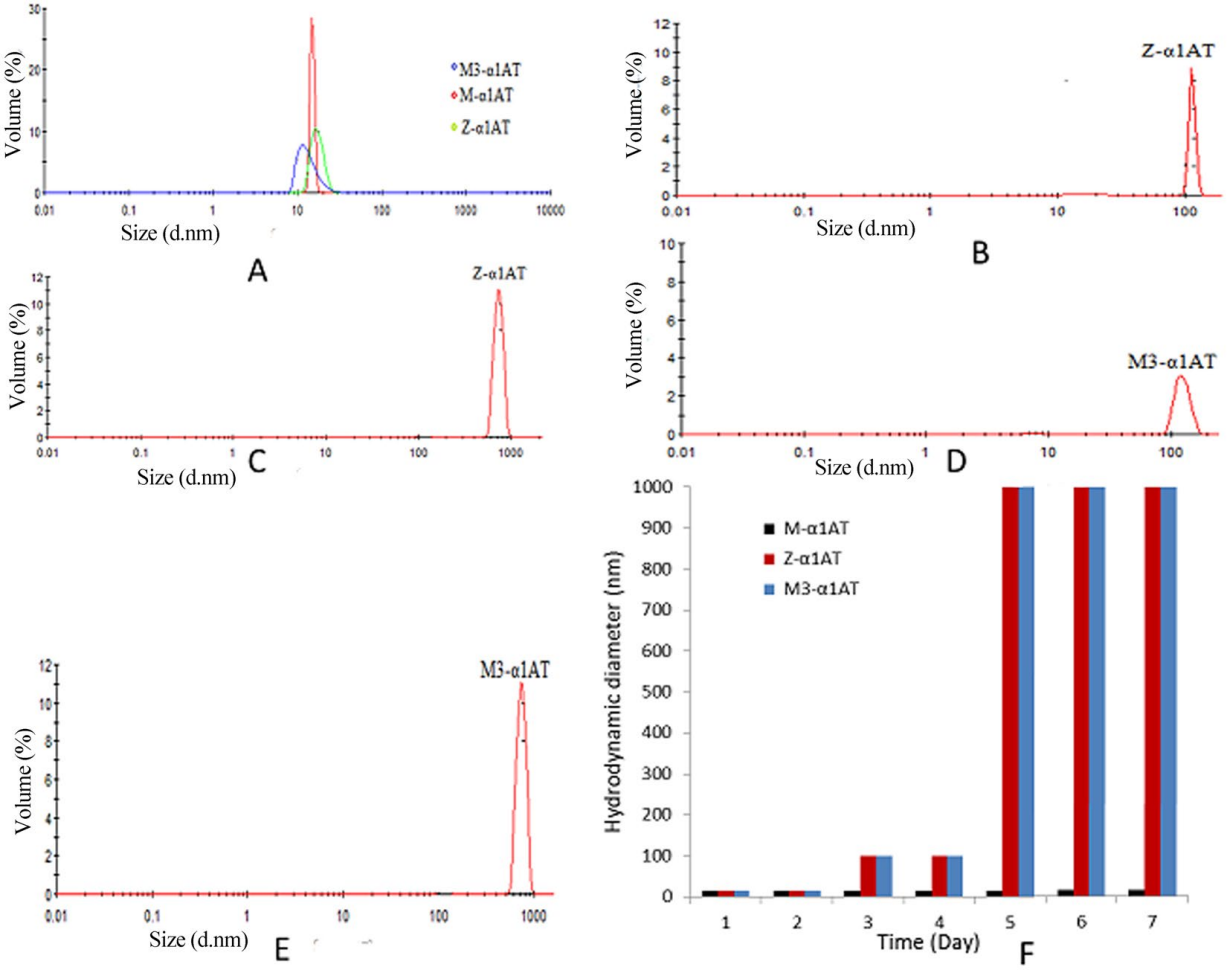
Both the α1AT variants (Z-α1AT and M3-α1AT) are capable of forming aggregates. Hydrodynamic diameter measurements of freshly prepared α1AT proteins (M-α1AT, M3-α1AT and Z-α1AT) showing a diameter of 15 nm **(A)**. Hydrodynamic diameter measurement of Z-α1AT proteins in a concentration and time-dependent manner showing the formation of aggregates of varied size from 100–1000 nm in diameter **(B,C)**. Hydrodynamic diameter measurement of M3-α1AT proteins in a concentration and time-dependent manner showing the formation of aggregates of varied size from 100–1000 nm in diameter **(D,E)**. Graphical representation showing variation of hydrodynamic diameter with respect to time **(F)**.

α1AT is a metastable protein and any pathogenic mutation makes it susceptible to aggregation. Z-α1AT has been reported to form aggregates of varied size and therefore, different hydrodynamic radii. All the freshly purified α1AT proteins subjected to dynamic light scattering (DLS) assay were found to exist in a dimeric state (Fig. 1A).

### Both the variants are aggregation-prone compared to the wild-type over a period of time

Z-α1AT has an ability to form aggregates at 37 °C of varied size over a period of time^14^. We did a time and concentration dependent DLS assay on α1AT proteins that were kept strictly at 4 °C. We observed that both the α1AT variants (Z-α1AT and M3-α1AT) are capable of forming aggregates of varied size over a period of time at 4 °C (Fig. 1B–E) excluding M-α1AT, which retained its dimeric form during this period. Native PAGE also corroborated the evidence of aggregate formation (Fig. S1). The Fig. 1F shows the graphical representation of the change of hydrodynamic diameter of α1AT proteins with respect to time.

### All α1AT proteins behave differently upon trypsin treatment

α1AT proteins showed a pronounced difference in the cleavage pattern upon treatment with trypsin (Fig. S2A–C). A prominent cleavage pattern was observed for the α1AT variants (M3-α1AT and Z-α1AT) compared to M-α1AT. Since the concentration of trypsin used is very less, one cannot observe the trypsin‒α1AT covalent complex. To confirm that the multiple protein fragments of size less than 52 kDa are from α1AT, we treated a fixed concentration of α1AT proteins with a known quantity of trypsin and subsequently probed with α1AT antibody. It turns out that all the cleaved products arise from α1AT proteins (Fig. S2D).

### Z-α1AT has higher stoichiometry of inhibition

Our data suggests that Z-α1AT protein has a higher stoichiometry of inhibition (SI) compared to M-α1AT and M3-α1AT upon trypsin treatment (Fig. S2E). This result almost correlates with the data furnished previously^15^. Interestingly, we found that M-α1AT and M3-α1AT have same SI. The higher SI of Z-α1AT protein could be due to the 342 Glu → Lys substitution that breaks a salt-bridge between them. This drives an electrostatic repulsion between these two amino acids that prolong the exposure of the C-terminal domain, thereby leaving Z-α1AT highly accessible to trypsin-driven cleavage^3^.

### Secondary structure percentage varies among the α1AT proteins

**Figure 2:**
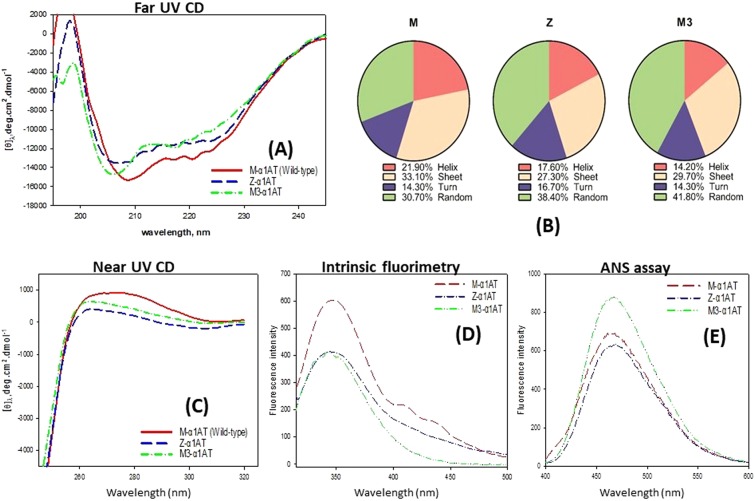
All the α1AT proteins vary in their secondary structures. (**A**) Far-UV CD spectrum of the α1AT proteins. The concentration of the α1AT proteins was 0.5 mg/mL and the readings were taken between 190‒250 nm. The path length of the cuvette used for far-UV measurements was 1.0 mm and 10 mm, respectively. All optical measurements were taken in the appropriate degassed buffer. The scan rate was 100 nm/min and each scan were an average of 3 accumulations. The optical spectrum of M-α1AT, Z-α1AT and M3-α1AT is shown by the red, blue, and green curve. **(B)** Fraction of secondary structures estimated *in-situ* by the instrument emiting the far-UV CD spectrum. The RMS-fit corresponding to the spectrum for M-α1AT, Z-α1AT and M3-α1AT are 18, 30, and 16, respectively. A lower value indicates a better fit. **(C)** Circular dichroism spectrum of the α1AT proteins in near-UV range. The concentration of the α1AT proteins was 0.5 mg/mL and the readings were taken between 240‒320 nm. The path length of the cuvette used for near-UV CD measurements was 1.0 mm and 10 mm, respectively. All optical measurements were taken in the appropriate degassed buffer. The scan rate was 100 nm/min and each scan were an average of 3 accumulations. The optical spectrum of M-α1AT, Z-α1AT and M3-α1AT is shown by the red, blue and green curve. **(D)** Intrinsic tryptophan fluorescence spectrum of α1AT proteins. The spectrum of M-α1AT, Z-α1AT and M3-α1AT is shown by the red, blue, and green curve. Fluorescence emission spectra (in triplicates) were measured in Perkin Elmer LS55 spectrofluorimeter in a 3 mm quartz cuvette at an excitation wavelength of 295 nm and emission from 300–500 nm. The concentration of α1AT was 0.5 mg/mL. **(E)** 8-aniline-1-naphthalene sulfonic acid (ANS) binding fluorescence assay. The optical spectrum of M-α1AT, Z-α1AT and M3-α1AT is shown by the red, blue, and green curve. The experiment was initiated by the addition of α1AT to the cuvette to give a final concentration of 0.1 mg/mL. The fluorescence changes of the ANS during α1AT polymerization were then measured by exciting at 360 nm and the fluorescence was recorded at 400‒600 nm.

The far-UV CD spectra of M-α1AT and its variants (Z-α1AT and M3-α1AT) is shown in Fig. 2A. A general decrease in the secondary structure percentage in the variants (Z-α1AT and M3-α1AT) and corresponding increase in randomness relative to the M-α1AT was observed. A decrease in α-helical content was observed near the negative bands at 222 nm and 208 nm in Fig. 2A. The 222 nm minimum of Z-α1AT (blue) and M3-α1AT (green) spectrum was found to be shallower than the M-α1AT (red). The 208 nm minimum of Z-α1AT (blue) and M3-α1AT (green) spectrum was observed to be shifted to lower wavelengths relative to the M-α1AT (red), which reveals an increase in disorder. The spectrum also revealed a decrease in the β-sheet content of Z-α1AT (blue) and M3-α1AT (green) compared to M-α1AT (red). Taken together, the far-UV CD spectra suggest that the α1AT variants are conformationally more relaxed relative to M-α1AT with decreased α–helicity and β‒sheet content. The percentage change in the secondary structure of the α1AT proteins calculated from the far-UV CD spectra is shown in Fig. 2B. This matches reasonably with the computed secondary structure from the molecular dynamic trajectories listed in Table S2.

### Both the α1AT variants have similar tertiary structure compared to the wild-type

The near-UV CD spectrum of M-α1AT and its variants (Z-α1AT and M3-α1AT) is shown in Fig. 2C. The M-α1AT shows a broad peak (maximum fluorescent intensity) at 275 nm followed by M3-α1AT and Z-α1AT. The near-UV CD spectrum of the variants is more or less retained and is comparable to the M-α1AT. At the same time, the decrease in signal coupled with a blue shift, which is more remarkable for Z-α1AT protein, reflects a small change in the tertiary structure that is more likely because of 342Glu^−^ → Lys^+^ substitution which increases the solvent exposure, thereby giving a room to the polar environment around tryptophan-194. Z-α1AT is expected to induce a change that is likely to be more pronounced compared to M3-α1AT (376 Glu^−^ → Asp^−^). The fluorescence emission spectra of the M-α1AT and its variants (Z-α1AT and M3-α1AT) are shown in Fig. 2D. The M-α1AT showed maximum fluorescence intensity at 350 nm. The variants were found to be of equal intensity but 34% reduced relative to the M-α1AT. α1AT protein has two tryptophan residues located at position 194 (buried inside the α1AT), and 238 that is exposed. Interestingly, the peak shifts in the 200‒240 nm region for both the variants in Fig. 2A are also reflected in Fig. 2C, suggesting that the environment of variants is different from the M-α1AT. The side chain of tryptophan-194 is packed between β-sA and β-sB, which forms a core of the α1AT structure. Interestingly, tryptophan-194 is located near the RCL of α1AT. The difference in intrinsic fluorescence intensity arising from the tryptophans is of the following order M-α1AT > M3-α1AT ≈ Z-α1AT (Fig. 2D), suggesting that the tryptophan burial around the β-sA and β-sB is much more in M-α1AT, than both the variants, which have very similar tertiary structure in that region. This points to the fact that both the variants are more exposed to the polar solvent compared to M-α1AT, thereby leading to decrease in the fluorescence intensity by a process known as fluorescence quenching. Overall, the near-UV CD coupled with the intrinsic tryptophan spectra of α1AT proteins reveal that both the variants have almost similar tertiary structure compared to wild-type (M-α1AT).

### M3-α1AT has exposed hydrobhobic regions compared to M-α1AT

The ANS-binding assay reveals that the M3-α1AT has more hydrophobic regions exposed compared to Z-α1AT and M-α1AT, which have similar exposure Fig. 2E. Overall, this assay suggests that M3-α1AT is more likely to form aggregates due to exposed hydrophobic patches which are buried in case of M-α1AT and Z-α1AT.

### Molecular dynamics simulations revealed difference in α1AT proteins among each other

**Figure 3:**
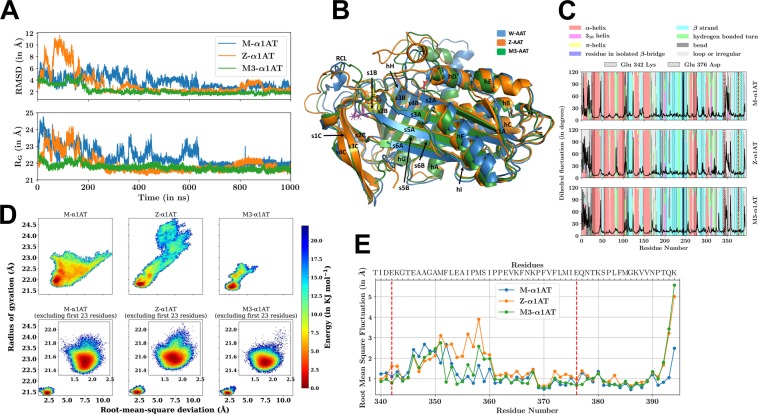
Results from the analysis of MD trajectories. (**A**) Backbone RMSD and R_G_ for different α1AT proteins. These trends are almost identical when these are evaluated using only C-α atoms or all atoms of α1AT proteins. **(B)** Aligned structures of the α1AT proteins in cartoon representation, each obtained from the deepest well in the respective free energy landscape. The mutation site 342 for Z-α1AT is shown in magenta and 376 for M3-α1AT is shown in red. All the secondary structure segments are labeled, and the nomenclature introduced here has been used throughout the text. **(C)** Fluctuation in backbone dihedral of various α1AT proteins and the distribution of the corresponding secondary structures in the protein. The locations of point mutation are marked with vertical dashed red lines. The proximal residues within 10 Å for each of the mutant sites are hatched as labeled. **(D)** RMSD and R_G_ free energy landscapes. All the subplots are in the same scale and the inset shows a zoomed view of the respective subplot. It can be seen that excluding the first 23 N-terminal residues removed the spread-out nature of the free energy landscapes (bottom row), and makes the landscapes similar in nature. **(E)** RMSF for various α1AT proteins restricted to C-terminal region (residues 340–394) from the trajectory between 700–1000 ns. The location of mutation at positions 342 and 376 is marked by vertical dashed red lines.

The first 23 N-terminal residues of α1AT proteins are highly flexible and undetected in all of the crystal structures available in the Protein Data Bank. Since, this region was modelled using Modeller and therefore all α1AT proteins showed different initial conformations. They required different time period to reach a stable conformation as can be seen from the backbone root mean square deviation (RMSD) with respect to the last frame (Fig. 3A, top panel). Results revealed that Z-α1AT has the highest RMSD during the first 200 ns of the trajectory (Fig. 3A). In the next phase (200–650 ns), M-α1AT showed a significantly greater RMSD compared to both the variants. The same trend was also observed in radius of gyration (R_G_), till 650 ns (Fig. 3A lower panel). From 650 ns onward all the α1AT proteins were seen relatively stabilized with RMSD limited to less than 3 Å. The modelled N-terminal segment is the primary cause of the large deviations in the initial part of the molecular dynamics (MD) trajectories and excluding this segment gave us a same free energy landscapes for all the α1AT proteins into consideration (Fig. 3D). For each α1AT, a representative structure from lowest free energy box of RMSD and R_G_ landscape have been aligned in Fig. 3B. The secondary structure assignment using DSSP showed that M-α1AT acquired an N-terminal helical structure at the earliest followed by M3-α1AT and Z-α1AT. The number of frames in which this N-terminal helix was observed in M-α1AT, M3-α1AT, Z-α1AT was approximately 4:2:1, suggesting that the M-α1AT has the most stable structure followed by M3-α1AT and Z-α1AT. The root mean square fluctuation (RMSF) graph looked similar for all α1AT proteins in most of the regions with a significant difference limited to the C-terminal segment, especially in the RCL (Fig. 3E). To have a greater clarity on the motion in this segment, we focused on comparative assessment of the trajectories between 700–1000 ns where the tertiary structure of the α1AT proteins got stabilized due to the formation of helical structures at the N-terminal segment. The differences in RMSF at the C-terminal region were further resolved by looking specifically between 350–394 amino acid residues. The result revealed distinct rigidity of M3-α1AT for the last four amino acid residues (P, T, Q, and K) followed by Z-α1AT and M-α1AT (Fig. 3E).

### The methionine-358 microenvironment is modulated by α1AT variants

**Figure 4:**
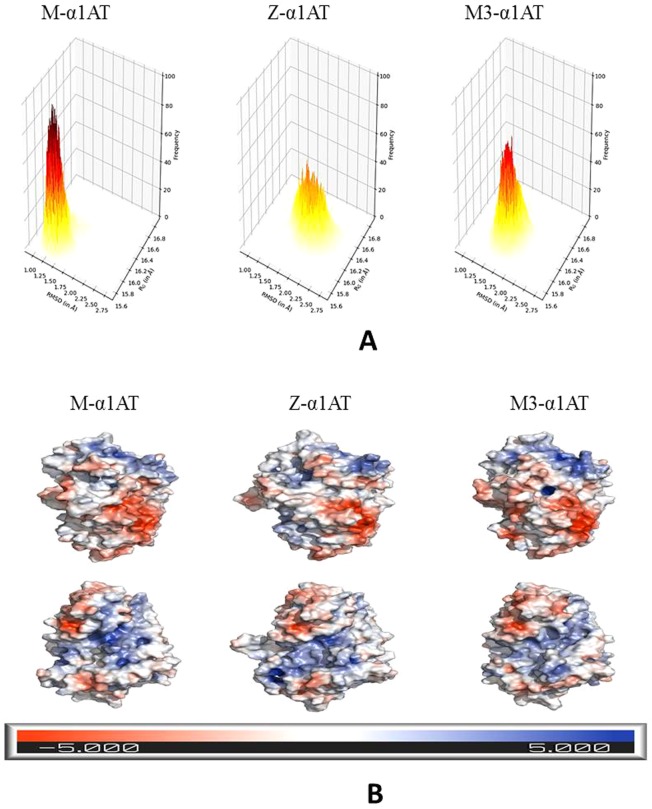
Conformational landscape and electrostatic potential of α1AT proteins. (**A**) 2D histogram of RMSD and R_G_ for the C-terminal region (340‒394) of α1AT proteins. The color scale is from yellow to brown according to the frequency value on the Z-axis. **(B)** Surface representation of α1AT proteins colored according to the electrostatic potential. The surfaces in each column are shown from two opposite orientations. The chosen structures belong to the deepest well in the free energy landscape for each α1AT protein. The electrostatic potential was calculated using Delphi software and represented by the coloring of the solvent accessible surface of the α1AT proteins based on the potential, and solvent radius above the surface, using PyMOL. Note: the marked distinction in the electrostatic potential on the surface is due to the mutation of a single residue between the proteins. The unit of the scale is in k_B_T/e.

A closer look at Fig. 3E reveals that Met 358, which forms a part of the RCL has its fluctuation mostly modulated by mutation at 342 in Z-α1AT, and 376 in M3-α1AT. Intriguingly, despite the difference in fluctuation, there is almost no difference in the secondary structure occupancies of the residues in the same time span. To have a better understanding of the conformational landscape of the α1AT protein for this duration, we looked at the population distribution of various conformational states in the α1AT proteins. The distribution of conformational states was found most constrained for M-α1AT followed by M3-α1AT and Z-α1AT (Fig. 4A). The M3-α1AT was observed to be comparatively more stable than Z-α1AT, although it also has a wide conformational well.

### Breach region is open in both the α1AT variants

It is well accepted that the relation between the RCL and the β-sA is vital for the enhanced lifespan of the metastable state of α1AT and any perturbation between them leads to the formation of aggregates^3^. The MD simulation revealed an open breach at the top of β-sA in the M3-α1AT. This is a unique hallmark of Z-α1AT that has been already reported^16^. The breach region is normally closed in the M-α1AT and is partially solvent exposed in case of Z-α1AT. It is the region where RCL coupled with deactivated protease first inserts in the main body of α1AT. This region comprises several highly conserved amino acid residues, in particular, tryptophan-194. The difference in solvent exposure to tryptophan-194 side chain in the wild-type (M-α1AT) and Z-α1AT simulation was observed by us and another investigator as well^16^. Due to the opening of the top of the β-sA masked by RCL, the solvent-exposed surface area of tryptophan-194 in wild-type (M-α1AT) and Z-α1AT, during the last 100 ns of the simulation, has been found to be 0.44 ± 0.15 nm^2^ and 0.78 ± 0.18 nm^2^. As far as the crystal structure of the wild-type α1AT is concerned, the carbonyl carbon of aspartate-341 forms a hydrogen bond with the side chain of tryptophan-194. Interestingly, the closure of the top of the β-sA in the breach region, early in the simulation of wild-type α1AT, results in the loss of this association. Conversely, in Z-α1AT, this hydrogen bond is maintained which increases the solvent exposure and polarity of the tryptophan-194. Our simulation results revealed the similar behavior in M3-α1AT.

### Electrostatic potential of α1AT proteins vary among each other

Electrostatics appears to play a key role in the perturbation that leads to protein aggregation. The residues with significantly different electrostatic potential among the α1AT proteins have been tabulated in Table S3. The distribution of the electrostatic potential on all the α1AT proteins is shown in Fig. 4B. The amino acid residue Thr 113, Ile 188, and Lys 335 transversely straddle sheet A located on strands β-s2A, β-s3A and β-s5A, respectively. These residues are juxtaposed underneath helix F and right at its C-terminal end and the subsequent random coil segment. The opening of β-s5A is important for the insertion of RCL into the main body of α1AT. Electrostatic perturbations appear to provide a similar perturbation through mean electrostatic potential modulation. Electrostatic potential calculations reveal that M3-α1AT is distinct from both M-α1AT and Z-α1AT. A similar electrostatic perturbation in Z-α1AT, that is distinct from M-α1AT and M3-α1AT, arises at Lys 290, Asp 341 and Lys 342, which are all-contiguous in space and located at one tethering end of the RCL. Electrostatic perturbation at tethering end of the RCL will undoubtedly perturb the equilibrium state of Z-α1AT compared to that found in M-α1AT. The 2D histogram of the C-terminal region (from amino acid residue 340‒394) of the α1AT proteins reveals the stability order as M-α1AT > M3-α1AT > Z-α1AT. The next set of spatially contiguous residues electrostatically found perturbed are Arg 223, Phe 227 and Asn 228 which lie in β-sC adjoining the RCL on one end and β-sB on the other. Interestingly, the RCL also becomes a part of sheet β-sC. Therefore, any electrostatic perturbation in this region is likely to affect the RCL equilibrium directly and of β-sB with which it packs.

Singleton electrostatic perturbations at methionine-351, that has the polarizable nature of the Sulfur in its side chain, may directly affect the reactive center equilibrium and the effects are likely to be magnified. The other noted perturbation at Ala 60 that connects the β-sA may aid in its perturbation during an aggregation process.

## Discussion

α1AT is an abundant circulating antiprotease in our blood. Its levels are elevated in inflammatory condition and are primarily meant to neutralize the elastase secreted by neutrophils at the site of inflammation. Different types of point mutations in α1AT gene like Z-α1AT has been extensively implicated in the reduced serum α1AT levels that may lead to COPD. The normal serum α1AT ranges from 0.9–2.0 g/L^17^. However, *Abcams* α1AT ELISA kit reports the normal serum level from 1.0‒3.6 g/L (Ab108798). The normal level of serum α1AT may vary from one region to another. The Kashmiri population mostly resemble ethnically and phenotypically with Iran and Iraq^18^; therefore, one would expect a genetic diversity in the normal serum α1AT levels. M3-α1AT (376Glu > Asp) is largely being considered as a normal variant of α1AT. Few investigations based on epidemiological data have reported an increased frequency of M3-α1AT in COPD, Alzheimer’s disease, chronic hepatitis, and demyelinating disease^11^. Our study also revealed an increased frequency of M3-α1AT (p < 0.0001) in Kashmiri population. The prevalence of M3 heterozygotic and homozygotic forms with the M, S, and Z variant background is not extensively reported. However, a study has reported the occurrence Q_0Madrid_ mutation in an M3 background, contributing to the pathogenicity. Keeping the above background into consideration, no study has identified how M3-α1AT could possibly play its pathogenic role in the development of different types of diseases including COPD.

The newly synthesized α1AT protein is enzymatically active in its metastable form (3). The exposed reactive center loop (RCL) of α1AT gets cleaved upon its interaction with proteases like neutrophil elastase and trypsin. This triggers a major irreversible structural rearrangement coupled with a decrease in free energy of α1AT^4^. Two models have been proposed to date to understand the mechanism of the α1AT polymerization. The first one proposes that RCL of the one α1AT instead of hopping into its main structure enters into the α1AT of another and the second models suggest the insertion of both RCL and β-s5A of α1AT molecule into another α1AT, also known as β-hairpin domain swap mechanism^19^. Both the proposed mechanisms led to the formation of a dimer that supports one of our experiments. It is interesting to note that in either of the events, the secondary and tertiary structure of the α1AT is maintained^19^. This is the main reason why Z-α1AT polymer accumulation in the ER of hepatocytes evades from unfolded protein response, a response generated by the cell to bring back the normal cellular homeostasis^20,21^. The widely accepted model of pathological α1AT polymerization is one in which the RCL of one α1AT anneals between β-s3A and β-s5A of another. Few investigations support this model. The first one is that RCL mimicking synthetic peptides have been shown to anneal between β-s3A and β-s5A, thereby, blocking α1AT polymerization and another one came from the fluorescence spectroscopy^22,23^.

The newly proposed mechanism of α1AT polymerization suggests that polymerization is preceded by the accumulation of a partially unfolded form (M*) in which β-s3A and β-s5A are separated that lead to the formation of a dimer to trigger polymerization cascade. The 15 nm diameter of all the freshly purified α1AT proteins calculated using DLS revealed their dimeric nature and progressively reflected an increase in aggregation ability for both the variants at 4 °C over a period of time except M-α1AT, which was still found in its dimeric state during the same time period.

Our results are contrary to the results furnished by Zhou *et al*.^24^ and others^25,26^ who have demonstrated that dimer formation is the minimum requirement to trigger and propagate α1AT polymerization. In this study, we observed that the dimeric nature of α1AT was retained by the wild-type at 4 °C over a period time compared to other two variants (M3-α1AT and Z-α1AT) that formed aggregates of varied size in the same time-period. Our results strictly point that both the variants can form aggregates of varied size even at 4 °C without any denaturant. It could also be possible that they might have formed a dimer in the bacterial expression cells prior to protein purification. It is important to note that α1AT synthesized by hepatocytes is secreted out in the circulation in glycosylated form, so the presence of monomeric α1AT in human blood is quite possible. The same holds true for α1AT protein synthesized by the yeast as well.

Our biochemical assay performed on α1AT proteins revealed that they behave differently upon their interaction with trypsin. More importantly, both the variants of α1AT (Z-α1AT and M3-α1AT) gave rise to multiple peptide fragments of less than 50 kDa in size compared to M-α1AT with the increasing concentration of trypsin. Therefore, one would suggest that both the variants are not in a native three-dimensional structure and have some exposed regions that provide a docking site for trypsin to bind compared to M-α1AT.

As far as our spectroscopic data is concerned, far-UV CD spectra of α1AT variants (Z-α1AT and M3-α1AT) revealed increase in randomness and conformationally more relaxed relative to M-α1AT. The percentage change in the secondary structure of the α1AT proteins calculated matches reasonably well with the computed secondary structure from the molecular dynamic trajectories. Additionally, the near-UV CD and intrinsic tryptophan spectra of α1AT proteins reveal that Z-α1AT and M3-α1AT have almost similar tertiary structure compared to wild-type M-α1AT. The ANS-binding assay reveals that the M3-α1AT has more hydrophobic regions exposed compared to Z-α1AT and M-α1AT. Overall, one would suggest that M3-α1AT is more likely to form aggregates due to exposed hydrophobic patches which are buried in case of M-α1AT and Z-α1AT. It is interesting to note that Met 351 and Met 358 are extremely critical for the antiprotease activity of α1AT^27^. Oxidation of either of the methionine in α1AT causes loss of its antiprotease activity that leads to α1AT dysfunction and is believed to be one of the possible mechanisms by which cigarette smoke can lead to emphysema.

The methionine 358 is susceptible to convert into methionine sulfoxide by oxidants like cigarette smoke, thereby rendering it much less potent anti-protease. The criticality of methionine-358 in maintaining the meta-stability of the C-terminal segment of α1ATs has been aptly revealed and the methionine to arginine mutation (α1AT_Pittsburg_ mutation) at this position is known to be lethal^28^. Normally, the 358^th^ methionine residue of the α1AT acts as bait for elastase. Likewise, the 393rd arginine residue of the antithrombin III acts as bait for thrombin and thereby regulates the blood coagulation cascade. This substitution switches over the anti-elastase activity of the α1AT to antithrombin activity, leading to fatal blood disorder^29^. Our results also indicate that methionine-358, which forms a part of the RCL have its fluctuations highly modulated by Z-α1AT, followed by M3-α1AT. All the results from our studies point towards an unusual behavior of M3-α1AT that resemble to some extent with the Z-α1AT.

## Conclusion

Our study revealed that M3-α1AT protein remarkably differ from the wild-type (M-α1AT) and has an ability to form aggregates like Z-α1AT. Furthermore, molecular dynamics simulation exploited for this study revealed that C-terminal region of M3-α1AT has high fluctuations compared to M-α1AT. However, further experiments are warranted to understand the mechanism/s by virtue of which M3-α1AT can contribute to the pathogenesis of COPD and other disorders as well.

## Materials and Methods

### Study

A case-control study was conducted with the main aim to find an association exon5 SNP (376Glu^−^ → Asp^−^) of the *SERPINA1* gene and the development of COPD patients in Kashmir valley. The research work was started following approval by the Board of Research Studies of University of Kashmir and the study protocol clearance by the ethical committee of the Government Medical College, Srinagar (34/ETH/GMC/ICMR). The study was conducted in accordance to the Declaration of Helsinki principles and Global Initiative for Chronic Obstructive Lung Disease (GOLD), 2014 revised guidelines^30^. All subjects gave informed and written consent to participate in the present study.

### Standard characteristics of study participants

A total of 230 ethnic-Kashmiris comprising of COPD cases (N = 110) and healthy subjects (N = 120) were studied from April 2014 to July 2015. Blood samples of COPD patients were collected from Government Chest Disease Hospital, Srinagar. Approximately, 2 mL of venous blood was taken from COPD patients (aged ≥40 years). Following inclusion criteria was strictly followed for COPD cases: (i) wheezing (high-pitched whistling sound), and/or cough along with the expectoration lasting more than three-months once a year (ii) Ratio of forced expiratory volume in one second to forced vital capacity (FEV1/FVC) < 70% and FEV1 < 80% on spirometry prior to salbutamol (bronchodilator) administration. Those COPD patients were excluded from our study who had a history of bronchial asthma, interstitial lung disease, lung carcinoma, active respiratory tract infection, nephropathy, seasonal influenza, allergy, and tuberculosis as revealed by the high-resolution computed tomography and chest radiography. A total of 40 such cases were disqualified from the present investigation in accordance to the GOLD guidelines. Overall, a total number of 70 COPD cases consisting of 21 females and 49 males were studied further. The controls comprising of 120 individuals with no history of COPD or for that matter any other serious ailments were included in the present study. DNA from the venous blood was collected from all the 120 individuals that served as a control for the present investigation. The controls were matched with the COPD cases separately for age (±5 years), gender, place of dwelling (rural/urban), smoking practice and ethnicity so as to reduce the confounding consequence of several significant factors. Fifteen healthy individuals were excluded from the study that had raised C-reactive proteins, post-surgical patients, and those who had any sign of inflammation strictly following the GOLD guidelines. Overall, 105 healthy individuals comprised of 65 males and 40 females were studied further.

### Patient data collection

The environmental factors, clinicopathological features, and demographic variables were personally collected and evaluated by discussing personally with the COPD patients and/or their caretakers. The information gathered were age, gender, place of residence, ethnicity, smoking practice, liver pathology, and the family history of COPD. The spirometry data of individual COPD cases was collected from the hospital. Additionally, relevant information from the controls was also collected. All the subjects or their custodians were properly informed about the present study. The willingness to participate in the present study was recorded from individual subject through a predesigned questionnaire.

### Genomic DNA extraction

Approximately, 2 mL of the venous blood sample was drawn from each participant in accordance to our established protocol^13^. The blood was collected in ethylene diamine-tetra-acetic acid-coated vials. The samples were kept in −80 °C until further processing. The phenol-chloroform DNA extraction method was strictly followed to isolate the genomic DNA from individual sample, respectively^31^. The purity (λ_260_/λ_280_ ratio) and the concentration (ng/µL) of the extracted DNA samples was carried out by using Bio-Rad’s *Nandrop™*. The DNA samples that had λ_260_/λ_280_ (purity ratio) falling within 1.80–1.91 range were used subsequently and those with λ_260_/λ_280_ ratio <1.80 were processed further to achieve desired purity ratio. Genomic DNAs were also qualitatively assessed by electrophoresing them in 0.8% agarose gel prepared in 1X TAE (Tris-acetate ethylenediaminetetraacetate).

### Polymerase chain reaction (PCR) for the amplification of exon 5 of the *SERPINA1* gene

A 494 bp size amplicon that encompasses 268 bp region of exon5 of the *SERPINA1* gene was amplified with the following set of primers (Pf:- 5′GTGACAGGGAGGGAGAGGAT3′ and Pr:-5′CTGTTACCTGGAGCCCACAT3′) under initial denaturation at 95 °C for 3:00 min, cyclic denaturation at 95 °C for 0:30 min, annealing at 62 °C for 0:30 min, cyclic extension at 72 °C for 0:45 min, and final extension at 72 °C for 7:00 min^32^.

### Genotyping

All possible variations in exon 5 of the *SERPINA1* gene of the COPD patients and controls was assessed through the direct sequencing method of the PCR products at SciGenom Private Limited, Kakkanad, Cochin, Kerala, India-682037 (http://www.scigenom.com).

### Cloning and site-directed mutagenesis

The wild type α1AT (M-α1AT) was cloned into the pET30a bacterial expression vector equipped with His tag at both N and C-terminal end. Next, primer set was designed to introduce Z mutation into the wild Mα1AT–pET30a construct to generate Zα1AT–pET30a.

### PCR reaction condition for site-directed mutagenesis to generate Zα1AT–pET30a construct

6652 bp recombinant Z-α1AT-pET30a was amplified with the following set of primers (Pf:-5′GCTGTGCTGACCATCGAC**AAA**AAAGGGACTGAAGCTGCT3′ and Pr: 5′AGCAGCTTCAGTCCCTTT**TTT**GTCGATGGTCAGCACAGC3′) under initial denaturation at 95 °C for 3:00 min, internal denaturation for 95 °C for 0.30 min, 65 °C for 0:30 min, 72 °C for 5:00 min, and final extension 72 °C for 10:00 min.

### PCR reaction condition for site-directed mutagenesis to generate M3α1AT–pET30a construct

6652 bp recombinant M3α1AT–pET30a was amplified with the following set of primers (Pf:-5′TTTGTCTTCTTAATGATTGA**C**CAAAATACCAAGTCTCCCCTC3′ and Pr:-5′GAGGGGAGACTTGGTATTTT**G**GTCAATCATTAAGAAGACAAA 3′ under initial denaturation at 95 °C for 3:00 min, internal denaturation for 95 °C for 0:30 min, 67 °C for 0:30 min, 72 °C for 5:00 min, and final extension 72 °C for 10:00 min.

### Large scale purification of α1AT proteins from *E. Coli. BL21-DE*

α1AT protein purifications were done as mentioned previously by Levina *et al*.^33^ with certain modification (5 M Urea added to dissolve the inclusion bodies).

### α1AT protein quantification

α1AT proteins were quantified by performing Bradford assay. The dilution of the samples was done in Milli-Q® water and mixed with equal volume of Bradford reagent. The known concentration of BSA was used as a standard. Samples were incubated at room temperature in dark for about 10 minutes and absorbance was recorded at 595 nm (*Thermo Scientific spectrometer*).

### Western-blot

#### Verification of α1AT protein and His tag in the recombinants

α1AT protein and His-tag verification was done by probing with anti-α1AT and anti-His antibody.

### Preparation of trypsin

The molecular weight of trypsin is 23.3 kDa. 0.25 mg of trypsin was dissolved in 1 mL of MQ-water. 0.25 mg/mL of trypsin (10.7 µM of trypsin) was used as a stock solution to prepare different concentrations of trypsin.

### Preparation of 0.54 µM α1AT proteins

0.5 mg/mL (9.61 µM) of α1AT proteins were used as a stock to prepare 0.54 µM α1AT proteins in 100 µL of cleavage buffer as described previously^34^.

### Trypsin-antitrypsin cleavage buffer

Trypsin-antitrypsin cleavage was performed at 37 °C in 30 mM sodium phosphate buffer, pH 7.4, containing 160 mM NaCl, 0.1% (w/v) polyethylene glycol, and 0.1% (v/v) Triton X-100.

### Far-UV and near-UV circular dichroism spectroscopy

The far and near-ultraviolet (UV) circular dichroism (CD) spectrum of purified wild-type α1AT (M-α1AT) and other two variants (Z-α1AT and M3-α1AT) were measured at least three times in a J-810 (Jasco spectropolarimeter) machine coupled with a Peltier-type temperature controller at ACBR, University of Delhi, India-110007. Each spectrum of the α1AT protein was corrected for the contribution of its blank. The final concentration of the α1AT proteins was 0.5 mg/mL. The path length of the cuvette used for far and near-UV CD measurements was 1.0 mm and 10 mm, respectively. The CD machine was regularly calibrated with D-10-camphorsulphonic acid. The secondary structure estimation from the far-UV spectroscopy was calculated by Yang’s method^35^.

### Fluorescence spectroscopy

Fluorescence measurements were done using a Perkin Elmer-LS 55 spectrofluorimeter at ACBR, University of Delhi-India. Intrinsic tryptophan fluorescence of all α1AT proteins was measured in 20 mM sodium phosphate, 100 mM NaCl, 0.1 mM EDTA, and 0.1% (w/v) PEG 8000, pH 7.4, using an excitation wavelength (λ_excitation_ 295 nm) and detecting photons emission at 90° to the excitation beam. The emission spectra were recorded from 300‒500 nm. The final concentration of all the α1AT proteins was 0.5 mg/mL. The path length of the cuvette used for fluorescence measurement was 5.0 mm. All the essential background corrections were done.

### ANS-binding assay

A saturated solution of ANS (8-aniline-1-naphthalene sulfonic acid) was prepared in 50 mM Tris, 50 mM KCl with pH 7.4. The ANS solution was filtered through a 0.2-mm pore size filter. The stock solution (50 mL) was added to 500 mL of 20 mM sodium phosphate, 100 mM NaCl, 0.1 mM EDTA, and 0.1% (w/v) PEG 8000 with pH 7.4 in a fluorescence cuvette. The experiment was initiated by the addition of α1AT to the cuvette to give a final concentration of 0.1 mg/mL. The change in fluorescence when ANS was incubated with respective α1AT protein was measured by exciting with the light at 350 nm. The fluorescence was recorded at 400‒600 nm.

### Dynamic light scattering experiments

The size distribution of α1AT proteins was obtained using Zetasizer MicroV/ZMV 2000 (Malvern, UK) at ACBR, University of Delhi-India. All the measurements were done using an incident laser beam of 689 nm at a fixed angle of 90°. Fifteen measurements were recorded at 25 °C with an acquisition time of 30 seconds for each α1AT protein at 10% of sensitivity. The data was analyzed using Zetasizer software provided by the manufacturer to acquire hydrodynamic diameters. The concentration of all the α1AT proteins was from 0.1–0.5 mg/mL.

### Molecular Dynamics (MD) simulation and in silico analyses

The structure of wild-type (M-α1AT) was obtained from the Protein Data Bank (PDB ID: 3NE4)^36^. The mutations 342 Glu → Lys (Z-α1AT) and 376 Glu → Asp (M3-α1AT) were introduced *in silico* using PyMOL^37^ to generate Z-α1AT and M3-α1AT variants, respectively. The 23 N-terminal residues of α1AT missing in the PDB structure 3NE4 were modelled using MODELLER^38^. The MD simulations were performed in GROMACS using the CHARMM27 force-field with cmap, available in GROMACS^39,40^.

The structures were placed in their respective dodecahedral box with dimensions close to 10 nm × 10 nm × 7 nm. There were variations in box sizes for each α1AT due to the difference in the structures after introducing the N-terminal region of it. Each box was solvated with water and the extended simple point charge model was used in the simulations. Na^+^ and Cl^−^ ions were added to neutralize the charge on the proteins while maintaining a final concentration of 0.1 M NaCl. The systems for M-α1AT, Z-α1AT, and M3-α1AT had 64722, 64728 and 68805 water molecules in the box, respectively. Periodic boundary condition was applied in all three directions. Particle-mesh Ewald (PME) and Verlet list was used in the simulations, as well as the default leap-frog integrator. The short-range electrostatic cut-off and short-range and van der Waals cut-off were both set at 1.2 Å, while the Fourier spacing was 0.12. Energy minimization was performed using the steepest descent algorithm with a step size of 0.01 kJ mol^−1^. The systems for M-α1AT, M3-α1AT and Z-α1AT converged to a maximum force below 1000.0 kJ mol^−1^nm^−1^ in 735, 682, 792 iterations, respectively. This was followed by equilibration for 100 ps in canonical (NVT) ensemble and for 100 ps in isothermal-isobaric (NPT) ensemble. Finally, the production run was for 1 µs, in which the frames were saved every 10 ps yielding 100,000 frames for analysis. The equilibration as well as final simulations was done at 300 K. The fluctuation in the dihedral angles (ɸ and ψ) for each residue was evaluated from the trajectory with the help of MDTraj python package^41^. The hydrogen bonds made by the mutated residues with rest of the molecule were calculated as well. The secondary structure throughout the trajectory was calculated using the GROMACS utility do_dssp, which uses the DSSP software behind the scenes^42,43^. The total and average propensity of various α1AT protein segments were calculated based on the amino acid propensities^44^. A conformational ensemble was selected across free energy landscape. The electrostatic potential around the molecule in each conformation was calculated using Delphi^45–47^. It uses a grid-based algorithm, which gives the electrostatic potential calculated at each grid point^46,47^. This was visualized by coloring the solvent accessible surface based on the value of the potentials (calculated earlier using Delphi) one solvent radius above the surface in PyMOL. The potential on each atom was calculated by obtaining its location within the grid and taking the average of the potential at corner grid points of the cell in which the atom resided. The potentials of atoms in each residue were averaged to get the value per residue. The difference in potential for Z-α1AT and M3-α1AT from the Wild-type (M-α1AT) was evaluated as well. To find the electrostatically perturbed residues; mean (µ) and standard deviation (σ) of the electrostatic potential was calculated for each residue. A range of the electrostatic potential values was created for each residue by taking the µ ± σ. The overlap between the ranges was calculated and converted into a percentage by taking the overlap range divided by the total range and the whole multiplied by 100. The comparisons were done for each pair of residues in the three proteins.

## Supplementary information

Supplementary information.
