# Supplementary material for: Aggregation of M3 (E376D) variant of alpha1- antitrypsin
Source: Sci Rep. 2020 May 19;10:8290. doi: 10.1038/s41598-020-64860-1 (PMC7237413; doi:10.1038/s41598-020-64860-1)
Supplement: Supplementary file 1 — Supplementary information. [file 41598_2020_64860_MOESM1_ESM.pdf]

Supporting Information

Aggregation of M3 (E376D) variant of alpha1 antitrypsin

Arif Bashir[1], Younis M. Hazari[1], Debnath Pal[3], Dibyajyoti Maity[3], Samirul Bashir[1], Laishram R. Singh[4], Naveed N. Shah[2], and Khalid M. Fazili[1*]

[1]Department of Biotechnology, University of Kashmir, Srinagar 190006, India,
[2]Department of Chest Medicine, Government Medical College, Srinagar 190006, India,
[3]Computational Data Sciences, Indian Institute of Science, Bengaluru 560012, India,
[4]Dr B.R. Ambedkar Center for Biomedical Research, University of Delhi, Delhi 110007, India

Running title: Aggregation of M3-α1AT

*To whom correspondence should be addressed

Khalid Majid Fazili
Professor
Department of Biotechnology
University of Kashmir-190006
fazili@kashmiruniversity.ac.in
bashir.aarif@gmail.com
Tel. (+91) 9419003881
Fax. (+91) 1942403610

Keywords: COPD: chronic obstructive pulmonary disease, α1AT: alpha1 antitrypsin, CD (circular dichroism) spectroscopy, molecular dynamics (MD) simulations, DLS: dynamic light scattering

Supporting Information

Figure S1. Native-PAGE of all α1AT proteins showing aggregation behaviour over 7 days of time.

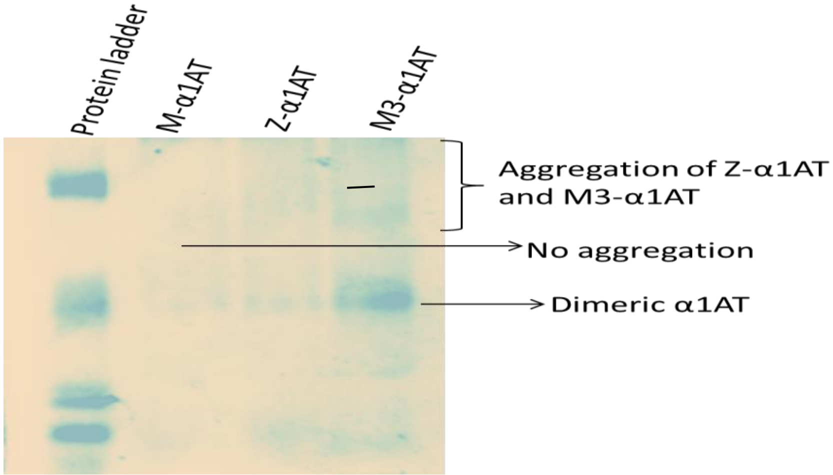

Figure S1

Figure S2. All α1AT proteins behave differently upon trypsin treatment.

(A-C). 12% SDS-PAGE of 0.54μM α1ATs (M-α1AT, Z-α1AT and M3-α1AT) without and with 0.06 μM trypsin incubated at 37°C for two minutes. Lane 1 represents multicolor protein ladder. Lane 2 represents a mixture with only 0.06 μM trypsin. Lane 3–5 represents undigested M-α1AT, Z-α1AT, and M3-α1AT. Lane 6–8 represents 0.06 μM trypsin treated M-α1AT, Zα1AT, and M3-α1AT.

(D). Western blot of 0.54μM α1ATs (M-α1AT, Z-α1AT and M3-α1AT) without and with 0.06 μM trypsin incubated at 370C for two minutes. Lane 1 represents multicolor protein ladder.

Lane 2 represents a mixture with only 0.06 μM trypsin. Lane 3-5 represents undigested M-α1AT, Z-α1AT, and M3-α1AT. Lane 6-8 represents 0.06 μM trypsin treated M-α1AT, Z-α1AT, and M3-α1AT.

(E). The inhibitory properties of α1AT proteins tested against trypsin on 12% SDS-PAGE. Lane 1 represents multicolor protein ladder. Lane 2 is loaded with 0.54μM M-α1AT + 0.010 μM trypsin, Lane 3 with 0.54μM M-α1AT+ 0.015 μM trypsin, Lane 4 with 0.54μM Z-α1AT+ 0.010 μM trypsin, Lane 5 with 0.54μM Z-α1AT+ 0.015 μM trypsin, Lane 6 with 0.54μM M3-α1AT+ 0.010 μM trypsin, and lane 7 with 0.54μM M3-α1AT+ 0.015 μM trypsin.

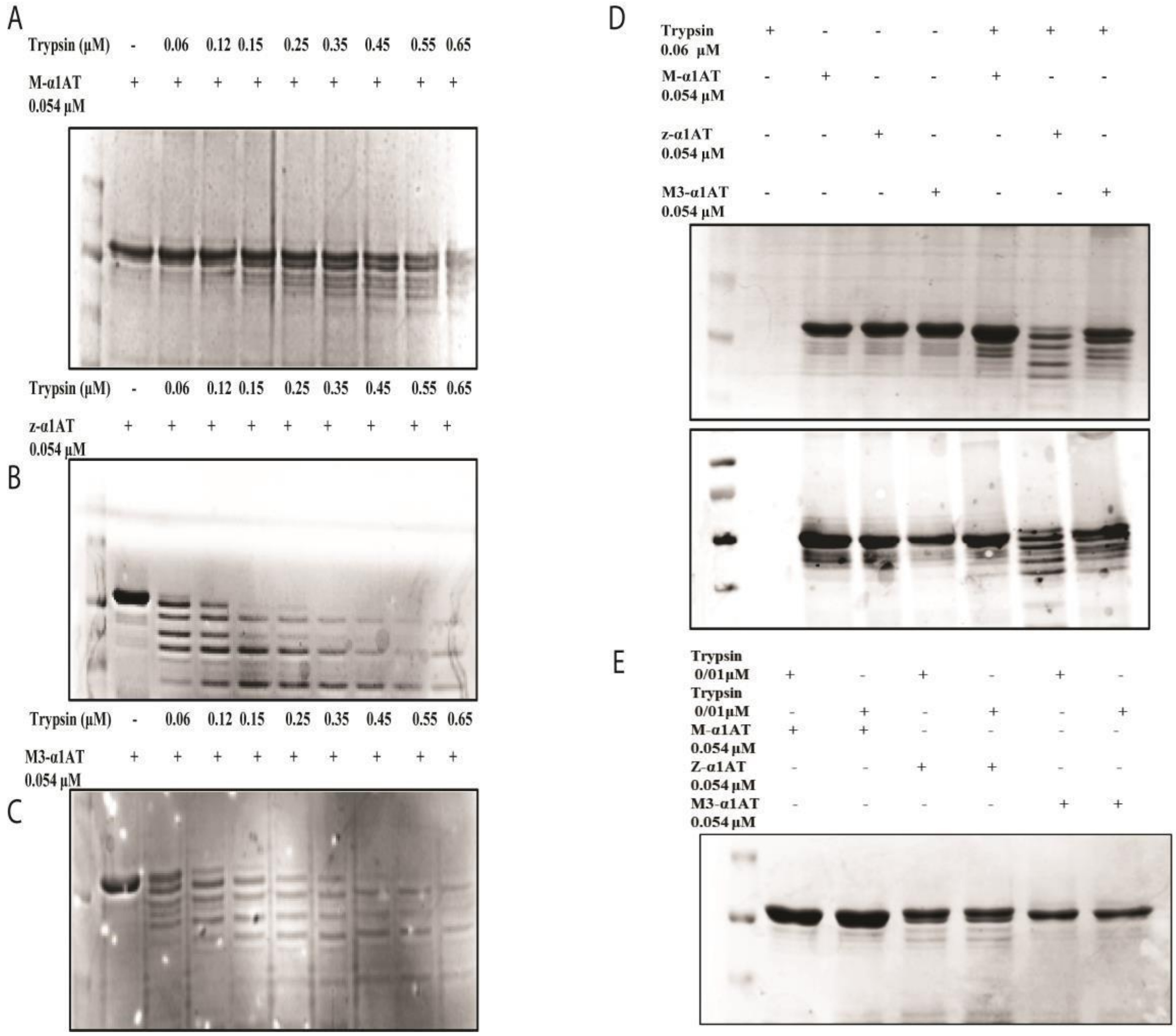
A
Trypsin (µM) - 0.06 0.12 0.15 0.25 0.35 0.45 0.55 0.65
M-α1AT 0.054 µM + + + + + + + + +
Trypsin (µM) - 0.06 0.12 0.15 0.25 0.35 0.45 0.55 0.65
z-α1AT 0.054 µM + + + + + + + + +
B
Trypsin (µM) - 0.06 0.12 0.15 0.25 0.35 0.45 0.55 0.65
M3-α1AT 0.054 µM + + + + + + + + +
C
D
Trypsin 0.06 µM + - - - + + +
M-α1AT 0.054 µM - + - - + - -
z-α1AT 0.054 µM - - + - - + -
M3-α1AT 0.054 µM - - - + - - +
E
Trypsin 0/01µM + - + - + -
Trypsin 0/01µM - + - + - +
M-α1AT 0.054 µM + + - - - -
Z-α1AT 0.054 µM - - + + - -
M3-α1AT 0.054 µM - - - - + +

Figure S2

Supporting Information

Table S1. Association of 376 A/C single nucleotide polymorphism with various demographic variables, environmental factors, clinicopathological parameters, and family history in COPD cases.

Characteristics	N=70	AA<br>14 (20.00%)	AC<br>41 (58.57%)	CC<br>15 (21.43%)	Odds ratio 95%CI, P value	χ2; P value (Overall)*
**Gender**						
Male	49 (70.00%)	10 (71.42%)	27 (65.85%)	12 (80.00%)	AC 1.29 (0.34 – 4.88); p = 0.7551	1.0635;
Female	21 (30.00%)	04 (28.57%)	14 (34.14%)	03 (20.00%)	CC 0.62 (0.11 – 3.47); p = 0.6816	0.5875
**Dwelling**						
Rural	45 (64.28%)	10 (71.42%)	22 (53.65%)	13 (86.66%)	AC 2.15 (0.58 – 8.01); p =0.3497	5.660;
Urban	25 (35.71%)	04 (28.57%)	19 (46.34%)	02 (13.33%)	CC 0.38 (0.05 – 2.53); p = 0.3898	0.0607
**Smoking status**						
Ever	42 (60.00%)	09 (64.28%)	22 (53.65%)	11 (73.33%)	AC 1.55 (0.44 – 5.44); p =0.5470	1.905;
Never	28 (40.00%)	05 (35.71%)	19 (46.34%)	04 (26.66%)	CC 0.65(0.13 – 3.19); p = 0.6999	0.3857
**Hemoptysis**						
Yes	25 (35.71%)	04 (28.57%)	18 (43.90%)	03 (20.00%)	AC 0.51(0.13 – 1.90); p = 0.3614	3.121;
No	45 (64.28%)	10 (71.42%)	23 (56.09%)	12 (80.00%)	CC 1.60(0.28 – 8.90); p = 0.6816	0.2099
**PFT**** =70%	52 (74.28%)	12 (85.71%)	28 (68.29%)	12 (80.00%)	AC 2.79(0.54 – 14.30); p = 0.3040	1.984;
<70%	18 (25.71%)	02 (14.28%)	13 (31.70%)	03 (20.00%)	CC 1.50 (0.21 – 10.65); p = 1.000	0.3707
**Obstructive jaundice**						
Yes	29 (41.42%)	03 (21.42%)	19 (46.34%)	07 (46.66%)	AC 0.29(0.07 – 1.30); p = 0.1245	2.885;
No	41 (58.57%)	11 (78.57%)	22 (53.65%)	08 (53.33%)	CC 0.31(0.06 – 1.59); p = 0.2450	0.2363
**Family history**						
Yes	44 (62.85%)	09 (64.28%)	28 (68.29%)	07 (46.66%)	AC 0.83 (0.23– 2.99); p = 1.0000	2.215;
No	26 (37.14%)	05 (35.71%)	13 (31.70%)	08 (53.33%)	CC 2.05 (0.46– 9.14); p = 0.4621	0.3303

* The values in bold indicate significant results. CI: confidence interval.

** PFT (pulmonary function test) done before the salbutamol nebulization to COPD patients.

Supporting Information

Table S2. Secondary structure distribution from DSSP calculation on the trajectory between 700 ns and 1000 ns.

	**M-α1AT**	**Z-α1AT**	**M3-α1AT**
**Structured**	0.66	0.63	0.63
**Helix**	0.30	0.27	0.29
**β-Sheet**	0.28	0.28	0.28
**Turn + Bend**	0.2	0.2	0.19
**Coil**	0.22	0.25	0.24

Table S3. Residues with significantly different mean electrostatic potential in the 700–1000 ns molecular dynamics trajectory.

**Residue position**	**Percentage overlap in mean electrostatic potential distribution**		
**M-α1AT**	**M-α1AT-Z-α1AT**	**M-α1AT-M3α1AT**	**Zα1AT- M3-α1AT**
60-Ala*	91.7	0.0**	0.0**
351-Met*	20.7	2.0	81.6
113-Thr	76.8	5.3	0.0**
188-Ile	88.0	17.1	4.1
335-Lys	92.8	0.0**	19.8
223-Arg	0.0**	0.0**	99.9
227-Phe	3.5	97.5	6.1
228-Asn	0.0	67.2	32.5
290-Lys	28.3	48.3	0.0**
341-Asp	0.0**	76.1	0.0**
342-Glu	0.0**	75.3	0.0**

* Residues shown in groups are contiguous to each other except for 60-Ala and 351-Met.
**Residues with zero overlap of mean electrostatic potential mean significant perturbation.
